# Transition to a New Cancer Care Delivery System: Opportunity for Empowerment of the Role of the Advanced Practice Provider

**DOI:** 10.6004/jadpro.2012.3.1.4

**Published:** 2012-01-01

**Authors:** Ruth McCorkle, Constance Engelking, M. Tish Knobf, Mark Lazenby, Marianne Davies, Rebecca Sipples, Ellyn Ercolano, Catherine Lyons

**Affiliations:** From Yale School of Nursing, New Haven, Connecticut; Smilow Cancer Hospital at Yale–New Haven, Connecticut; CHE Consulting Group, Inc., Mt. Kisco, New York; White River Junction Veterans Affairs Medical Center, White River Junction, Vermont; Dartmouth College, Hanover, New Hampshire

## Abstract

The purpose of the study was to obtain an in-depth understanding of the perceptions of advanced practice providers (APPs) with respect to their current roles in the context of the transition to a new cancer care delivery system, as well as factors that may influence their ability to practice at their level of training and education. Five focus groups were conducted with 15 APPs (11 nurse practitioners, 4 physician assistants). Data were collected by a recorder at each focus group. Four investigators reviewed the data from each group for accuracy and to generate an initial set of codes. Codes were compared across reviewers until consensus was reached and final themes were agreed upon. The mean age of the participants was 43.5 years (range: 27 to 63 years). The APPs practiced for an average of 11 years (range: 1 to 27 years), with a mean of 6.5 years in oncology (range: 1 to 11 years). Six themes were generated from the data related to the APP role during the transition to a new oncology care system: experiencing role tension, facing communication barriers, seeking mentorship, dealing with fragmented care, recognizing the need for professional growth, and navigating a new system. Our findings may inform administrators about the role of the APP in quality care delivery. These findings may empower APPs to practice to the full scope of their training and educational preparation, thereby facilitating their goals for professional development.

Recent major advances in diagnosis, treatment, and care delivery demonstrate the ever-evolving health-care system in the United States. This constant evolution places greater demands on staff members, altering their roles and requiring them to attain new competencies. This is especially true in cancer care, as significant therapeutic discoveries and the introduction of new care delivery systems have not only increased care complexity but also shifted the majority of care to the outpatient setting.

The specialty of oncology provides a great sense of professional and personal satisfaction for many professionals. However, today’s health-care system challenges nurse practitioners and physician assistants: rapid changes in basic science and cancer therapy and increased complexity of care and acuity of patients (both inpatient and outpatient) may lead to frustration and cause stress as the gap between the challenges of the system and their desire to practice to the fullness of their scope widens (Ackerman, Mick, & Witzel, 2010).

Many academic oncology practices incorporate into the care team nurse practitioners and physician assistants, clinicians we refer to collectively as advanced practice providers (APPs). The use of APPs in oncology practices is a viable solution to address workforce issues and the increased needs of more acute patients receiving increasingly complex care without compromising on quality and efficiency. However, little information is available to understand the roles, responsibilities, and practice patterns of the APPs in these settings (Hinkel et al., 2010; Towle et al., 2011).

In 2010, Smilow Cancer Hospital (SCH) at Yale New Haven and Yale Cancer Center (YCC) transitioned from a multisite cancer care delivery system that was both university- and hospital-based to a new, state-of-the-art, 14-story comprehensive cancer center building. The new hospital includes all services (inpatient and outpatient) and specialties (surgery, radiation, medical oncology, and support services).

Along with the facility transition, there was also a transition to a new philosophy of cancer care for SCH and YCC built on the value of patient- and family-centered care. The care delivery system in this new philosophy of cancer care is built around 12 multidisciplinary disease-based teams (breast, lung, gastrointestinal, hematologic, and others). Clinical care and clinical research are in a dynamic relationship. Clinicians conduct research and at the same time deliver care that is infused with the core values of communication and coordination and centered on patients and families (see Figure 1).

**Figure 1:**
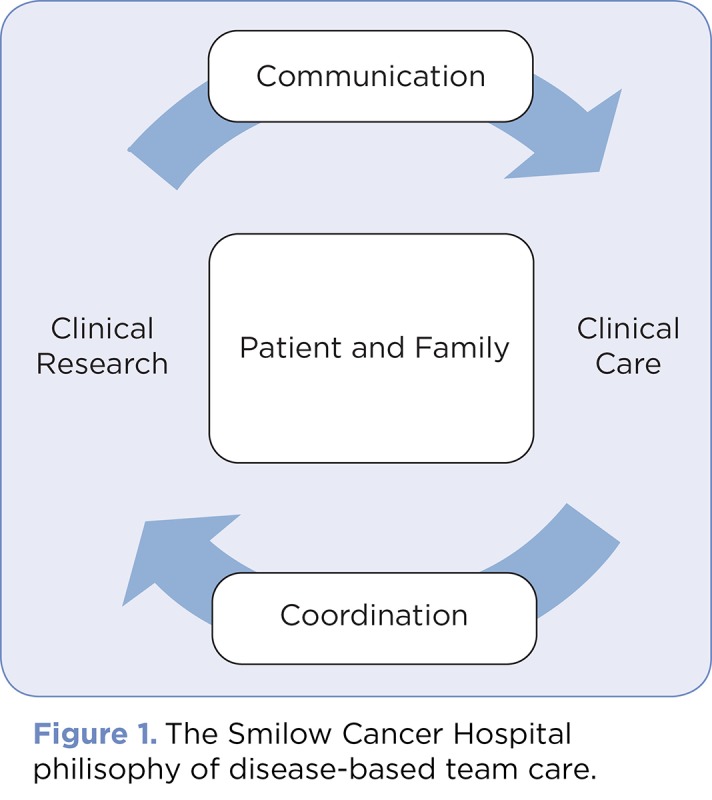
Figure 1. The Smilow Cancer Hospital philisophy of disease-based team care.

While the structure within the system is evolving, efforts have been made to recognize APPs as integral to its disease-based team approach. Bringing these providers together to practice within a uniform structure required that services and support systems be centralized for accessibility and productivity. The disease-based teams are multidisciplinary and the new structure places the APP in a central role of being a link among members of the disease-based team. True to the philosophy of cancer care from which the structure arose, the primary role of APPs is to be key communicators who coordinate clinical care in a context in which clinical research is conducted and patients and families are central. The goal is for APPs to perceive themselves as key members of the disease-based teams and true partners with their physician colleagues. Conversely, other members of the disease-based team need to perceive APPs as key contributors to quality patient- and family-centered cancer care (see Figure 2).

**Figure 2:**
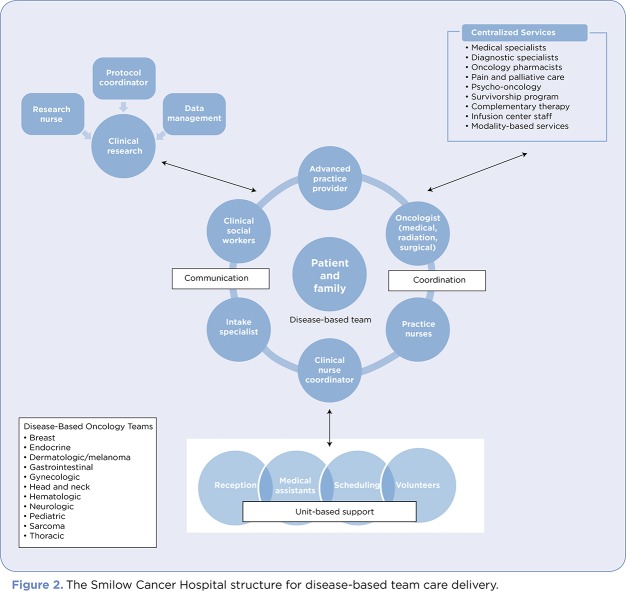
Figure 2. The Smilow Cancer Hospital structure for disease-based team care delivery.

In the context of the lacuna of literature on APPs roles in an academic cancer care setting and the opportunity of the transition to a new cancer hospital, the purpose of this study was to describe APPs’ perceptions of their current roles and to identify factors that may influence their ability to practice within the full scope of their practice. The overall goal was to engage APPs and empower them to practice within their disciplinary perspective and to the full scope of their educational preparation, licensure, and clinical skills. Data presented in this paper are part of a larger study focused on identifying potential barriers to delivering quality cancer care vis-à-vis the structure of the care delivery system in order to assist the SCH leadership in prioritizing areas for improvement to maximize the impact of APPs’ roles on clinical outcomes.

## METHODS

**SAMPLE AND INTERVIEWS** 

As there are limited data on APPs’ perceptions of and actual role implementation in a comprehensive cancer center, a qualitative research approach using focus group methodology (Krueger & Casey, 2000) was chosen to understand APP roles. Focus groups are a valid method for examining APPs’ role perceptions, and they are particularly useful in facilitating expression of opinions and solutions for improving practice. The APPs were informed about the project, detailing the confidentiality of their participation, in a general information session prior to initiating the groups. Participants were recruited via email. A general information email was sent to all 32 APPs reminding them of the focus groups and schedule. A second email was sent a week prior to each scheduled group session, with a third reminder email sent the day before and requesting an RSVP. The project received an exemption from review by the Yale University’s Human Subjects Research Committee because there were no risks to participants. Demographic, education, and clinical data were obtained from each participant.

Between October and November 2010, five focus groups lasting between 60 and 90 minutes were conducted at times convenient for participants. All groups were facilitated by the first author (R.M.), an advanced practice nurse with extensive research and educational experience in oncology. A dedicated recorder collected data at each group session and recorded comments verbatim. At least one PA was in all five focus groups. Focus group size ranged from 3 to 4 participants, with a total of 15 participants; one PA attended two groups.

The format of each focus group was similar. Group dialog was free-flowing though loosely structured around a set of predetermined questions in five categories: (1) clinical practice, both current and ideal roles, (2) communication, (3) standards of care, (4) clinical research participation, and (5) mentorship and professional development. Questions were organized first to engage participants, then to explore their role in specific areas, and finally to close the session. The facilitator introduced the reason for the focus group, indicating that the aim was to learn the views of APPs regarding their current roles, including factors that facilitated or impeded their ability to do their jobs. Participants were initially asked a broad question, "Can you describe your current role?" Additional interview questions elicited information on participants’ perceptions of the daily responsibilities and their ability to work with others in the delivery of care, as well as the structure of the system of care to facilitate their work.

**DATA ANALYSIS** 

Discussions in the focus groups were documented by a recorder who did not participate in analysis of the findings. Initially, the written descriptions were read in their entirety by four independent reviewers (authors R.M., C.E., M.D., and R.S.); this procedure yielded 131 significant statements from which initial codes were developed. The reviewers compared codes in a joint session until agreement on codes and their meanings was reached. As new concepts were identified, codes were expanded or consolidated into different conceptual categories (Mays & Pope, 2000) and then categorized into six themes that characterized the data. To further ensure credibility of the data analysis, two general meetings were held with the entire APP practice group to review the themes and confirm the interpretation of the data.

## RESULTS

**PARTICIPANT CHARACTERISTICS** 

Out of 32 APPs, 15 (47%) responded to our request. This included 11 NPs and 4 PAs with an age range from 27 to 63 years (mean: 43.5 years). All were licensed, and 12 (82%) held additional specialty certifications, e.g., oncology, adult, palliative care. Participants reported practicing in their APP role for a mean of 11 years (range: 1 to 17 years) with an average 6.5 years (range: 1 to 11 years) in oncology. The sample is described further in Table 1.

**Table 1:**
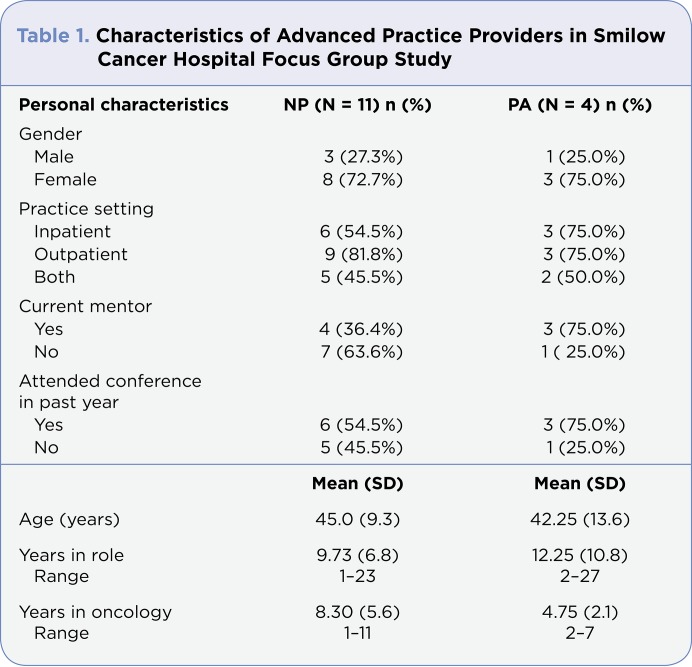
Table 1. Characteristics of Advanced Practice Providers in Smilow Cancer Hospital Focus Group Study

**THE APP ROLE IN THE TRANSITION TO A NEW CANCER CARE DELIVERY SYSTEM** 

Six themes were generated from the focus group qualitative data: experiencing role tension, facing communication barriers, seeking mentorship, dealing with fragmented care, recognizing the need for professional growth, and navigating a new system (see Table 2). Participant feedback is presented in italic type.

**Table 2:**
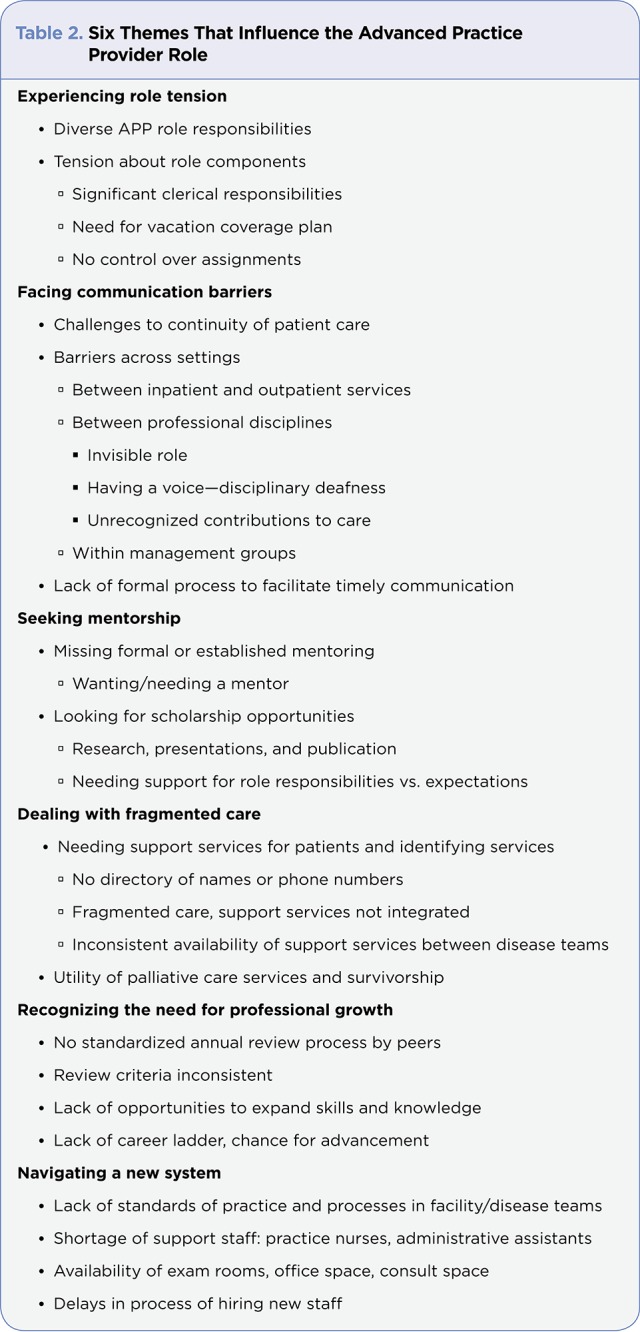
Table 2. Six Themes That Influence the Advanced Practice Provider Role

*Experiencing Role Tension*: Both NPs and PAs reported great variability in their roles across disease teams and between inpatient and outpatient settings. *"The workload/assignment is inconsistent, some have high patient volumes while others not so much…It would be better to be more evenly distributed."* All participants reported a high level of satisfaction in providing direct care to patients, but they also experienced considerable tension in responding to the demands of providing indirect care activities, such as answering telephone calls, scheduling appointments and procedures, and ordering prescriptions. They stated that clerical responsibilities diverted their attention from direct patient care. *"I spend most of my day on the phone, answering questions that a clerk could handle. It would be really helpful if we had a triage system so calls could be screened."*

Some reported that they felt they had minimal or no control over patient assignments in the clinic and often did not see returning patients; this process challenged their ability to provide continuity of care. *"A list of patients is posted on a corkboard. Providers see patients on a next available basis because we are always running behind. I may have no real working knowledge of the patient and his problems and may have to check with the physician, which may require waiting or interrupting the doctor."*

They reported that once a new position was posted, the process of hiring new APP staff was long and time-consuming, especially the length of time it takes to verify licenses and credentials before newly hired APPs can practice.

*Facing Communication Barriers*: The APPs felt that there were major barriers within the system that interfered with communication. They reported a lack of awareness of formal mechanisms for sharing information across services, especially between specialties. One major barrier to effective communication is the existence of three different documentation systems (electronic inpatient, electronic outpatient, and paper charts in selected clinics). While transition to a new integrated system is in progress, the current systems challenge everyday practice related to effective continuity of care for patients. "*The documentation systems are not compatible across services, especially between inpatient and outpatient and between medical oncology and radiation*." They realized many patients are often treated concurrently across services, and that although physicians were aware of the APPs’ recommended treatment plans for teaching and management of symptoms and treatment side effects, opportunities for APPs to participate in ongoing discussions with physicians to maximize patients’ comfort levels were limited.

Advanced practice providers in the outpatient clinics had no formal mechanism to alert them when one of their patients was admitted for an unscheduled visit to the hospital. "*You see a patient in the clinic, then later find out he was admitted and you didn’t know; it’s embarrassing. Some of these patients I’ve been seeing for a long time, and I have information about their care that might make a difference in their recovery.*" As they transitioned into the new facility, an expanding list of centralized supportive services was evolving. They reported that they did not know who the primary contact was and where to find the right person for common referrals, including nutrition, palliative care, social work, and psychiatry.

*Seeking Mentorship*: The APPs reported that the demands of their current roles were so time-consuming that they had few opportunities for professional development. "*I would love to be able to carve out time to participate in research and publications. I have a lot of good ideas about how to improve patient care.*" They felt that their workload also interfered with their ability to bring forth clinical questions for discussion with team members or to pursue research questions with nursing and medical faculty. The majority reported that they did not have a mentor; one participant reported that he would have no idea who that person would be and how one would use a mentor.

Thirty percent reported attending a national or regional conference within the past year. Others reported that they had difficulty obtaining release time and coverage; they did not request time off for professional development, despite the reality of needing CEU/CME credits to maintain certification. All respondents reported a desire to have ongoing opportunities for professional development, including conferences, seminars, research projects, fellowships, and formal education. "*I should be investing more in keeping current with the literature, but other things are going on, mainly hectic clinics. It all adds up to become a burden at times.*"

*Dealing With Fragmented Care*: Advanced practice providers in the focus groups reported that access to the institutional supportive care resources (social work, nutrition, physical therapy, psychiatry, and palliative care) varied across disease teams. They felt that there was no easy way to identify who to call, where these resource persons were located in the new hospital, and how to effect a timely referral. Several respondents stated that they had difficulty finding such resources to help when patients were at their clinic appointments. "*There are problems with trying to reach the appropriate individual to discuss a consult. There is no one place to find telephone numbers and email contacts, no directory for consults, and the paging system is poor. I cannot reach people when I need them*." Another stated that patients in the clinic could be seen by palliative care staff in the hospital, but was unaware of services in the clinic. Another reported that she had several patients who needed to be evaluated by a psychiatrist, but similarly there was coverage only on the inpatient units. "*We need a psycho-oncology service; I am forced to refer patients for mental health services in their own community. When I do that, there is no continuity and I don’t receive feedback as to what is recommended.*"

*Recognizing the Need for Professional Growth*: The respondents reported that there is no standardized annual review process to receive feedback about their performance. Several reported that they had never had an annual review; those who had, were evaluated by an administrator or physician. The majority reported that they would prefer to be evaluated by a peer or manager with consistent criteria across settings, such as the competencies developed by the Oncology Nursing Society (ONS, 2007).

Advanced practice providers reported that there are limited opportunities to advance within their practice compared to others. They mentioned that there is a clinical ladder for advancement of staff nurses who also have opportunities to participate in an oncology nursing review course, but nothing similar for NPs. "*I wonder why there is a formal support structure for the Nursing Department but not for the APPs, when it feels like there should be. I feel like we aren’t valued.*" They acknowledged that physicians have routine seminars and grand rounds providing them with information on advances in basic science and cancer management, thus facilitating their recertification with continuing education credits. Likewise, APPs need regular structured activities they can attend and opportunities for ongoing education to keep them current such as latest research for management of treatment side effects, clinical practice standards to prevent complications and maximize outcomes.

*Navigating a New System*: The APPs also described tensions associated within the new hospital system that they felt interfered with their abilities to provide quality care. They expressed frustration with moving into a new, unfamiliar physical space. They reported their perception that there were inadequate support services, such as staff to answer telephones, respond to questions, and schedule procedures. In the ambulatory care areas, APPs had difficulty finding patients in examination rooms because they were unaware of systems to identify patient locations. Advanced practice providers shared that their clinic day is often disrupted by unpredictable urgent patient visits within their fixed clinic appointment schedules. "*If people are sick and need to come in, they will somehow be seen. But there’s no room, no chairs, and it disrupts the schedule but we see them.*" Inconsistent standards of practice were another concern. "*There are relatively few evidence-based standards of care/practice and there is a need to come together formally to create them as a basis for practice.*"

## DISCUSSION

Our findings provide preliminary data of how APPs in an academic cancer center perceived their current and desired roles as they transitioned to a new cancer care hospital. This transition offered the opportunity to examine APP role function and identify challenges for APPs in delivering care during an organizational transition. During times of transformative change such as this, APPs and other provider groups have a chance to exercise leadership in defining how they can best actualize their role to benefit patients. To capitalize on the possibilities, they need to determine how best to negotiate and advocate for themselves and, ultimately, for the patients and families in their care. Conducting focus groups is itself a strategy to raise awareness and stimulate action on the part of the participants. The focus group project served both to engage APPs in the process of clarifying their current roles and to empower them in the way that coheres with their disciplinary perspective, expertise, and scope—a role that can serve as an exemplar for APP role definition in cancer care. 

Kanter (1977) describes four organizational empowerment structures that closely match some of the major thematic areas we have identified: access to information, access to support, access to resources needed to do the job, and access to opportunities to learn and grow.

Access to information is access to knowledge of organizational decisions, policies, and goals. By informing APPs about how the decisions to evolve a new structure of care delivery were made, APPs would gain a sense of purpose and meaning in their roles and responsibilities in the newly formed teams. This sense of purpose and meaning would empower them to make decisions about their roles and how they would work together, including addressing the tensions within the system that they identified, and contributing to the organization’s goals for the new structure of care delivery.

Access to support encompasses feedback and guidance from a 360¡ view—from those who report to APPs, to those who work alongside them, and to those to whom they report. The emotional support, helpful advice, and hands-on assistance that others within the organization can provide from such 360¡ review would enable APPs to move beyond the communication barriers that present challenges to continuity of patient care. Indeed, such open lines of feedback and guidance would be the very mechanism for identifying and correcting future barriers and lacunae of formal processes of communication within the organization. As APPs stated in the focus groups, if the tensions around communication were addressed, the organizational philosophy of patient- and family-centered care embodied in the new structure of care delivery would be enhanced.

Access to resources includes the ability of APPs to access the materials and support services they need. In our study, APPs experienced role tension. This tension centered on support services and resulted from inadequate dissemination of a rapidly expanding supportive care service with a variety of new providers (e.g., psychiatrist, behaviorist). Collaborating with administration to develop strategies to identify the most recent additions to supportive care providers would enhance patient- and family-centered care during times of transition and reduce role tension among APPs.

The fourth structural factor, access to opportunity for mobility and growth, entails access not just to knowledge gained and CEUs earned but also a model of APP practice that supports their level of education and expertise. In the current climate with economic forces, demographic factors, and gaps in access to providers, it is more critical than ever to maximize the contributions of APPs to quality patient- and family-centered care (Fairman, Rowe, Hassmiller, & Shalala, 2011). Our findings indicate that structural empowerment during the planning stages of organizational transition may help providers integrate the transition and more effectively implement a new structure of care delivery. By engaging providers in dialog about current or expected transitions, administrators would facilitate discussions of goals and how to collectively work towards them (Nevidjon & Simonson, 2009).

Our findings also suggest that a major transition into a new hospital can prompt changes in providers’ attitudes and abilities to engage in the process. Advanced practice providers’ feedback was important in identifying gaps in APP knowledge about the new structure as well as APP perceptions of best practices. Their insights have further informed the development of staff education, communication strategies, and interventions to streamline clinical operations. Their feedback was also helpful in defining strategies for improving the APP role within the newly formed disease-based teams to enhance patient and family and professional satisfaction and in formulating the new structure of care delivery as illustrated in Figure 2.

These insights into the need for organizational involvement of APPs from the planning, transition, and rollout stages of major changes in facilities and care delivery structures may be useful to other oncology APPs, whether they work in large, multidisciplinary centers or small community settings. However, a few limitations are noted.

We chose to interview APPs because of the critical nature of their role in establishing successful multidisciplinary disease-based teams in a comprehensive cancer center. Although the focus on a single cancer hospital and small sample size is appropriate in qualitative research, additional research is needed to determine how APPs’ role transitions are handled and experienced among different providers. It should also be noted that the size of each focus group was never larger than four and may have limited their discussions. Our response rate was also limited to about half of the APP population; however, over 80% attended one of the two larger meetings to discuss the results. Additional work can help inform how individuals can best learn to manage the challenges that accompany transitioning cancer care during relocation of services while maintaining quality.

## CONCLUSION

Data from focus groups with 15 advanced practice providers (both nurse practitioners and physician assistants) identified the importance of each provider’s engagement and contribution to the transition to a new facility and a new philosophy of care. Six themes of internal and external role tensions were constructed from the data which appear similar to what Kanter (1997) identified. In this phase of the facility transition, the APPs have brought clarity to the vision of an APP role that fully actualizes their skills and knowledge. The next phase of our implementation plan is to formally establish an APP Council for Professional Development to facilitate becoming leaders in quality initiatives and expanding professional development opportunities (Eaton & Tipton, 2009; Melnyck & Fineout-Overholt, 2005).
